# Supplementary material for: RNA polymerase III transcription–associated polyadenylation promotes the accumulation of noncoding retrotransposons during infection
Source: Proc Natl Acad Sci U S A. 2025 Aug 6;122(32):e2507186122. doi: 10.1073/pnas.2507186122 (PMC12358842; doi:10.1073/pnas.2507186122)
Supplement: Supplementary file 1 — Appendix 01 (PDF) [file pnas.2507186122.sapp.pdf]

SUPPLEMENTAL INFORMATION
Supplemental Methods:

Cell lines and siRNA nucleofections
Cells were maintained in DMEM with 10% FBS and grown to 90% confluence. Cells were removed and washed once with Dulbecco's phosphate-buffered saline (DPBS) (Gibco). Nucleofections were done using the Neon Transfection System (Thermo Fisher). 2×10^6 cells were resuspended in 100 µL of buffer R, to which control non-targeting, Brf1, CPSF30, CPSF73, or Nudt21 pools of siRNA (ON-TARGETplus SMARTpool siRNA – Horizon Discovery) were added to a final concentration of 200nM. This was loaded into a Neon 100-µL pipette tip and Neon tube with 3 mL of buffer E2 with electroporation parameters set to 1,300 V, 20 ms, 2 pulses. Following electroporation, cells were plated in 10 mL of DMEM with 10% FBS in 10 cm TC-treated plates and were incubated at 37°C for 24 hours.

Chromatin immunoprecipitation (ChIP)
NIH3T3 mouse fibroblast cells plated in a 10-cm TC-treated dish were cross-linked in 1% formaldehyde in DPBS (Gibco) for 5 minutes at room temperature, quenched in 0.125 M glycine, and washed twice with DPBS (Gibco). Cross-linked cells were harvested and lysed with 1 ml ChIP lysis buffer (50 mM HEPES [pH 7.9], 140 mM NaCl, 1 mM EDTA, 10% glycerol, 0.5% NP-40, 0.25% Triton X-100) by rotation for 10 min. Nuclei were collected by centrifugation at 1,700 × *g* for 5 min at 4°C, washed once with ChIP shearing buffer (50 mM Tris-Cl, pH 7.5, 10 mM EDTA, 0.1% SDS), and then resuspended in 1 ml of ChIP shearing buffer. Chromatin was then sheared for 5 min using a Covaris S220 focused ultrasonicator at 140 power, a 5% duty cycle, and 200 bursts/cycle. Chromatin was spun at 15,000 × *g* for 5 min at 4°C, and the pellet was discarded. 40 µg of chromatin was incubated with 10 µg rabbit polyclonal anti-POLR3A (Abcam ab96328), anti-CPSF30 (Proteintech 15023-1-AP), or rabbit IgG (Abcam ab37415) overnight. 25 µl of mixed protein A and G Dynabeads (Thermo Fisher) were added, and the tubes were rotated for 2 hours at 4°C. Dynabeads were then washed with low-salt immune complex (20 mM Tris [pH 8.0], 1% Triton X-100, 2 mM EDTA, 150 mM NaCl, 0.1% SDS), high-salt immune complex (20 mM Tris [pH 8.0], 1% Triton X-100, 2 mM EDTA, 500 mM NaCl, 0.1% SDS), lithium chloride immune complex (10 mM Tris [pH 8.0], 0.25 M LiCl, 1% NP-40, 1% deoxycholic acid, 1 mM EDTA), and Tris-EDTA for 5 min each at 4°C with rotation. DNA was eluted from the beads using 100 µl of elution buffer (150 mM NaCl, 50 µg/ml proteinase K) and incubated at 50°C for 2 h and then at 65°C overnight. DNA was purified using an Oligo Clean and Concentrator kit (Zymo) and eluted in 50 µl of nuclease-free water. DNA was used for generating libraries for ChIP-seq.

ChIP sequencing and data analysis
Sequencing quality was assessed with FastQC, and all sequencing files were processed using HTStream with default settings for paired-end reads, including trimming adapter sequences, PhiX screening, "N" nucleotide trimming, and removing low-quality reads. The mm10, yeast (GCA_000146045.2), and MHV68 (U97553.2) genomes were manually indexed using the bowtie2-build command. Processed sequencing files were then separately aligned to the three genomes with bowtie2 (1). Processed sequencing files were then separately aligned to the three genomes with bowtie2 (1) using the following parameters: --maxins 1500, -k 25, --no-mixed, and --no-discordant to allow for multiple mapping locations per read. Allo (2) algorithm was used to redistribute multimapping reads, including those that map to highly repetitive Pol III-transcribed

genes. The resulting bam files were further filtered to discard any unmapped reads and supplementary alignments (samtools -F 2048), ensure reads are properly paired (samtools -f 2), and blacklist regions on the mm10 genome (bedtools intersect -v)(3, 4). Peak calling was performed with MACS2 (5) on Polr3A ChIP-seq data with input normalization and default settings except for setting --min-length to 150 to capture small Pol III-transcribed genes. Bedtools intersect -u was performed on the mm10 RepeatMasker (6) file and called peaks to identify the precise loci that were bound by Polr3A. Consensus Polr3A-bound genes were then identified using bedtools intersect between replicates with the -f 1, -r, and -u options. All consensus Polr3A-bound genes in the mock and MHV68-infected conditions were concatenated into one file for differential binding analysis with DiffBind/DESeq2 (7, 8) using spike-in normalization scale factors proportional to the total number of spike-in reads. Spike-in normalization was used to compare across different sample conditions. Differentially bound regions were defined as FDR<0.05 and a log2FC>1.5. Polr3A coverage correlation plots with B2 SINE ncRNA or pre-tRNA expression levels were generated by using the bedtools map command to identify the max (-o max) coverage value from a bedGraph converted BigWig file for the specified genomic regions.

ChIP-seq visualization

For Polr3A and CPSF30 ChIP-seq data in NIH3T3 cells, BigWig files were first generated with the bamCoverage command for each bam file from each sample mapped to the mm10 genome with the following parameters --binSize 10, --smoothLength 30, --extendReads, --centerReads, --normalizeUsing None, and --scaleFactor with their respective spike-in normalized scale factor (reciprocal of the ones used in DiffBind). CPSF30 ChIP-seq data in HeLa cells were similarly processed but were instead $\log_2$ input-normalized with the bamCompare command with the following changes in parameters: --scaleFactorsMethod None, --normalizeUsing RPKM, and –operation log2. Average BigWig file was then generated for each biological condition with the bigwigAverage command containing the -bs 10 option. The calculation for heatmap of Polr3A and CPSF30 coverage profiles was generated using the computeMatrix scale-regions command with the average BigWig files (-S), the mm10 blacklist option (-bl) for redundancy, --skipZeros, --missingDataAsZero, and the following options for visualization purposes: --beforeRegionStartLength 500, --regionBodyLength 300, --afterRegionStartLength 500. For Polr3A ChIP-seq data in NIH3T3 cells and CPSF30 ChIP-seq data in HeLa cells, the lists of regions were generated from Pol III-transcribed RepeatMasker genes that overlap with called peaks. All metaplots and heatmaps were generated using alignment files that were averaged from two replicates. ChIP-seq signal was plotted as a histogram with 10 bp bins for -500 to +500bp around the transcription start site (TSS) with 30 bins/gene (unless otherwise indicated). Each row of the heat map corresponds to the ChIP-seq signal from each Pol III gene from the metaplot above. Heat maps are ranked by Polr3A or CPSF30 ChIP-seq coverage values. For all IGV tracks, all alignment files were averaged across two replicates.

SAMBAR-Net architecture

Due to the repetitive nature of B2 SINE genes within a subfamily, a subset of the training and test sets could have highly similar sequences, which could cause the model to overfit quickly. To prevent overfitting, a simple DenseNet-like (9) architecture was developed with only three convolutional layers and two dense blocks – one between each convolutional layer – to reduce the number of parameters to train. Each convolutional layer contained 128 filters with a width of 10 base pairs. Batch normalization, ReLu activation, maxpooling (kernel size=5, stride=1), and

dropout (probability=0.3) layers were applied to each convolutional layer. Each dense block contained three convolutional layers, each with 128 filters with a width of 3 base pairs, padding of 2, and a growth rate of 32, in which each layer is directly connected to every other layer in a block. Each convolutional layer in a dense block had batch normalization and ReLu activation layers applied.

CNN training and evaluation

All settings used in TF-MoDISco were set to center the discoverable full-length nucleotide motif. These settings were consistent across all three B2 SINE subfamilies analyzed by the CNN model. Position frequency matrix (PFM) was extracted from TF-MoDISco and converted to a position weight matrix (PWM). Position frequency matrix (PFM) was extracted from TF-MoDISco and converted to a position weight matrix (PWM) by normalizing the frequencies to probabilities and dividing each probability in the matrix by the background frequency of nucleotides for the mm10 genome (A=0.29, C=0.21, G=0.21, T=0.29). PWMScan (10) was used to calculate PWMScores for expressed B2 SINEs as defined in the SINE-seq dataset (11). PWMScores reflect the strength of prediction for the binding site identified through TF-MoDISco. All settings were set to default when using PWMScan. Expressed B2 SINEs were then divided into quartiles based on their PWMScores.

Northern blotting

10 μg of total RNA was separated by electrophoresis on 8% TBE-Urea gels and transferred onto a Hybond N^+ membrane (Amersham). If RnaseH digestions were performed, 10 μg of total RNA was combined with 500 pmol of oligo(dT) primer in a 25 μl reaction, incubating at 65°C for 8 min, then adding 1 U of RnaseH (New England Biolabs), RnaseH buffer, and 40 U of Rnasin (Promega). Reactions were incubated at 37°C for 30 min, then terminated by adding 1 μl of 0.5 M EDTA (pH 8.0) and precipitating the RNA in 1ml of 100% ethanol before gel electrophoresis. Membranes were cross-linked at 2400 J/m^2 (UV Crosslinker, VWR). B2 SINE ncRNA was detected using the following digoxigenin-labeled (DIG) end-labeled oligo: GATGGTTGTGAGCCACCATGTGGTTGCTGGCA. Oligos were end-labeled using a DIG Oligonucleotide Tailing Kit (Roche). Gels were stained with 1 μg/ml Sybr Safe (Invitrogen) to detect 5.8S and 5S rRNAs as loading controls.

Primer extension

RNA was ethanol precipitated in 1 mL 100% EtOH, washed in 70% ethanol, and pelleted at 21,130 × *g* and 4°C for 10 min. Pellets were resuspended in 18 μL of 1X SuperScript III reverse transcriptase reaction buffer (SSIII-RT; Thermo Fisher) containing 1 μL of each 5′-fluorescein-labeled primer (10 pmol/μL). Samples were heated to 80°C for 10 min, followed by annealing for 1 h at 56°C. Then, 30 μL of extension buffer (1X SSIII-RT buffer, 40U Rnasin Rmase Inhibitor [Promega] 2 mM DTT, 1 mM dNTP, 1,000 U of SSIII-RT) was added, and extension was carried out for 1 h at 42°C. Samples were precipitated in 100% ethanol for 20 min at −80°C, and then pellets were briefly air dried and resuspended in 20 μL 1× RNA loading dye (47.5% formamide, 0.01% SDS, 0.01% bromophenol blue, 0.005% xylene cyanol, and 0.5 mM EDTA). Then, each sample was run on an 8% urea-PAGE gel for 1 h at 250 V. Gels were imaged on a Chemidoc imager (Bio-Rad) with fluorescein imaging capability. The relative induction of B2 SINE ncRNAs for each sample was measured as the ratio of the mean integrated intensity between 7SK RNA and B2 SINE ncRNA level using FIJI (12) was normalized to the mock-treated control.

Western blotting

Cells were washed with cold DPBS (Gibco) followed by lysis with radioimmunoprecipitation assay (RIPA) lysis buffer (50 mM Tris HCl, 150 mM NaCl, 1.0% [vol/vol] NP-40, 0.5% [wt/vol] sodium deoxycholate, 1.0 mM EDTA, and 0.1% [wt/vol] SDS, Roche cOmplete Mini EDTA-free protease inhibitor cocktail). Cell lysates were vortexed briefly, rotated at 4°C for 15 min, and then clarified by centrifugation at 21,000 × *g* in a tabletop centrifuge at 4°C for 10 min to remove debris. 30 μg of whole-cell lysate were resolved on 4% to 15% mini-PROTEAN TGX gels (Bio-Rad). Transfers to polyvinylidene difluoride (PVDF) membranes (Bio-Rad) were done with the Trans-Blot Turbo transfer system (Bio-Rad). Blots were incubated in 5% milk in TBS with 0.1% Tween 20 (TBS-T) to block, followed by incubation with primary antibodies. Washes were carried out with TBS-T. Blots were then incubated with HRP-conjugated secondary antibodies (Southern Biotechnology, 1:5,000). Washed blots were incubated with Clarity Western ECL substrate (Bio-Rad) for 5 min and visualized with a ChemiDoc imager (Bio-Rad).

Comprehensive identification of RNA-binding proteins (ChIRP) and mass spectrometry

~ 100 million NIH3T3 cells were infected with MHV68 at an MOI 5. 24 hpi cells were cross-linked with 1.1% formaldehyde for 15 min at room temperature. Crosslinking was then quenched with 0.125 M glycine for 5 min. Cells were rinsed again with PBS, scraped into Falcon tubes, and pelleted at 1000 xg for 5 min. The cell pellet was resuspended in 3 mL nuclei lysis buffer (50 mM Tris-HCl [pH 7.0], 10 mM EDTA, 1% SDS, protease cocktail inhibitor [Roche], and RNase inhibitor [Fermentas]) and rotated for 10 min. at 4°C. Cells were dounced10 times with a B-type pestle and separated into three 1 mL aliquots for sonication. Sonication was performed using a Covaris-focused sonicator. After sonication, chromatin aliquots were combined, and 9 mL of hybridization buffer (750 mM NaCl, 1% SDS, 50 mM Tris 7.0, 1 mM EDTA, 15% Formamide, protease inhibitor cocktail, and RNAse inhibitor) was added. 50 pmol of five separate 3′-TEG biotinylated probes were added to the diluted chromatin and rotated overnight for 16 h (Supplemental Methods). Streptavidin-magnetic C1 beads (Life Technologies) were washed three times in nuclei lysis buffer, blocked with 500 ng/μl yeast total RNA, and 1mg/ml BSA for 1 hr at room temperature, and washed three times again in nuclear lysis buffer before being resuspended in its original volume. One hundred microliters of washed/blocked C1 beads were added to the chromatin mixture and rotated for an additional 4 h at 37°C. Beads:biotin-probes:RNA:protein adducts were captured by magnets (Invitrogen) and washed five times with 10 mL wash buffer (2× SSC, 0.5% SDS). After the last wash, complexes were eluted by resuspending the beads in 500 μL G50 buffer (20 mM Tris-HCl, 300 mM NaCl, 2 mM EDTA, 0.2% SDS), The elution was split in half for isolation of protein and RNA. 50 μg/mL Proteinase K (Fermentas) was added to the fraction for nucleic acid isolation and incubated at 60°C for 1 h. The G50 buffer was separated from the beads, phenol-chloroform was extracted, and ethanol precipitated. RNA was extracted and analyzed by small RNA northern blotting. Protein was similarly isolated, except no Proteinase K was added. For mass-spectrometry, samples were provided to the University of California, San Francisco Mass Spectrometry facility for processing, trypsin digestion, and analysis by LC-MS.MS on a Thermo Scientific Velos Pro ion trap mass spectrometry system.

The five separate 3′-TEG biotinylated oligos used were:

LacZ oligo sequences: 1-CCAGTGAATCCGTAATCATG, 2-TCACGACGTTGTAAAACGAC, 3-AATAAGTTGGGTAACGCCAG, 4-AGGTTACGTTGGTGTAGATG, and 5-AATGTGAGCGAGTAACAACC

B2 SINE oligo sequences: 1-TAACCACTGAGCCATCTCTC, 2-
TGGAAGAGCAGTCAGGTGCT,
3-TGGGAATTGAA CTCAGGACC, 4- TTGTGAGCCACCATGTGGTT, and
5-GCATCAGATCTCATTACAGA

A

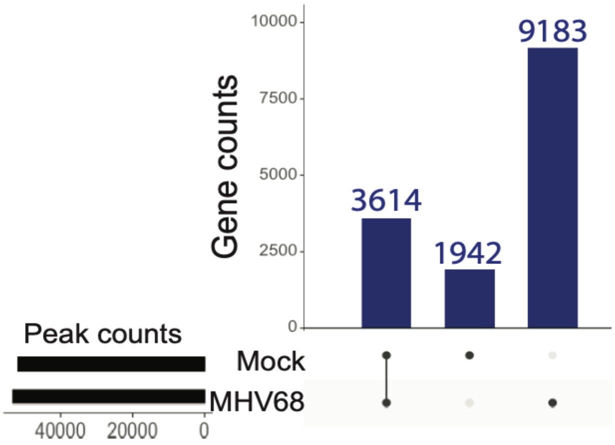

B

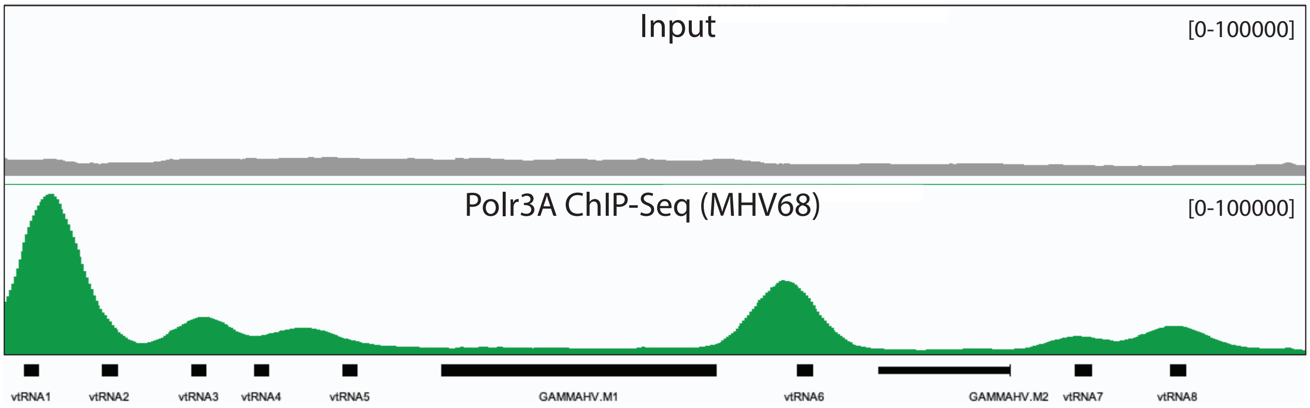

Figure S1. Recruitment of RNA Polymerase III to the MHV68 genome

(A) MACS2 (5) peak calling algorithm was applied to Polr3A ChIP-seq data from mock-treated or MHV68-infected (MOI of 5 for 24 hours) NIH3T3 cells to determine the number of Polr3A bound peaks across the murine genome. Peak positions were then overlapped with RepeatMasker (6) gene annotations to determine which subset peaks overlapped with Pol III transcribed genes. The upset plot shows the number of peaks in each sample condition on the left and plotted on the right is the number of Pol III transcribed genes overlapping with these peaks that are unique to mock-treated or MHV68-infected samples or are present in both samples. (B) Polr3A ChIP-seq coverage across the MHV68 genome from MHV68-infected samples. Alignment files were averaged from two replicates and visualized in the Integrative Genome Viewer (13). Y-axis maximum and minimum values are within brackets. Viral TMER genes (vtRNA1-8) are shown below as a reference.

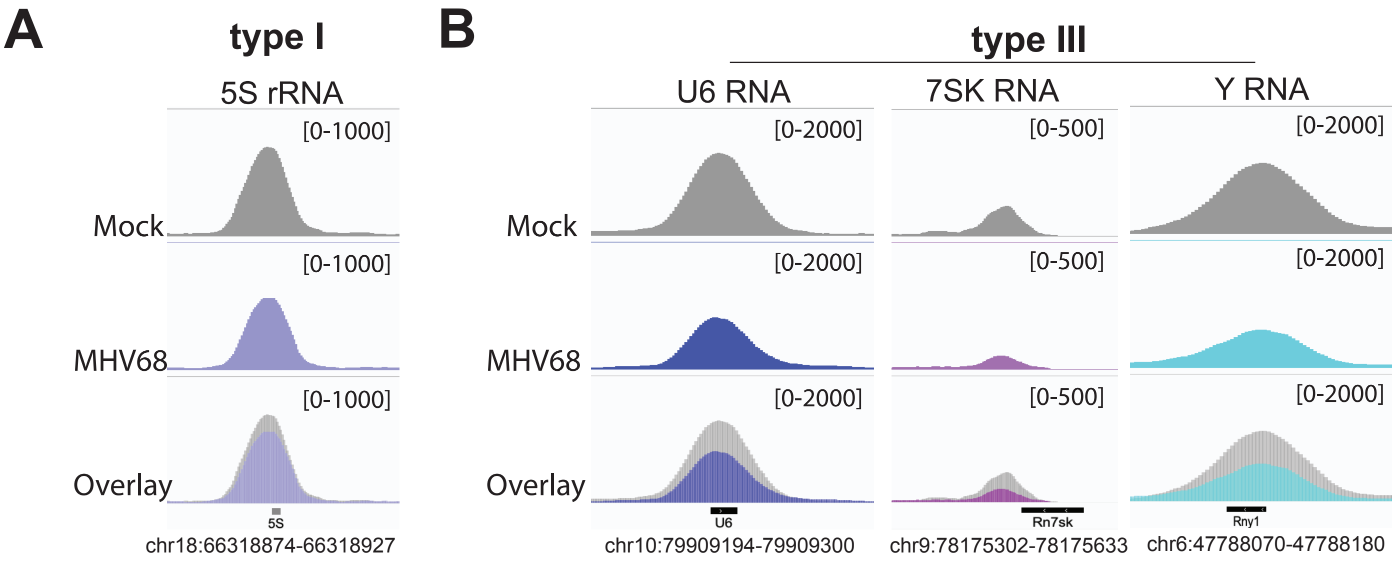

Figure S2. RNA Polymerase III binding to type I and type III promoters on the cellular genome during MHV68 infection

Polr3A ChIP-seq coverage across select promoter type I (A) and type III (B) genes from mock-treated or MHV68-infected samples. Alignment files were averaged from two replicates and visualized in the Integrative Genome Viewer (13). Y-axis maximum and minimum values are within brackets.

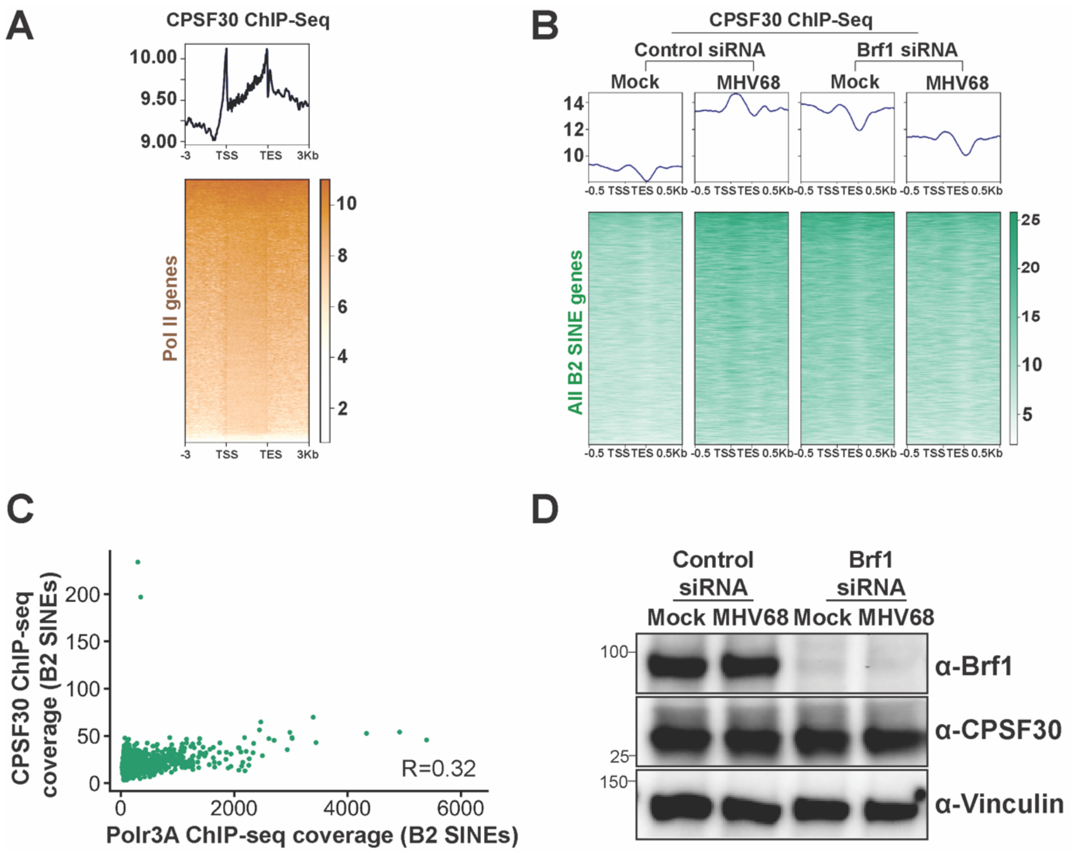

Figure S3. CPSF30-seq analysis at B2 SINE loci during MHV68 infection

(A) NIH3T3 cells transfected with control non-targeting siRNAs and harvested for ChIP-seq. The top panels are metagene plots displaying CPSF30 ChIP-seq signal across Pol II genes with the highest CPSF30 occupancy (n=10000). Alignment files were averaged from two replicates. ChIP-seq signal was plotted as a histogram with 10 bp bins for -3000 to +3000 bp around the transcription start site (TSS). In the bottom panel, each row of the heat map corresponds to the CPSF30 ChIP-seq signal for each Pol II gene from the metaplot above. (B) NIH3T3 cells transfected with control non-targeting or Brf1-targeting siRNAs were mock-treated or infected with MHV68 at an MOI of 5. At 24hpi, cells were harvested for ChIP-seq. The top panels are metagene plots displaying CPSF30 ChIP-seq signal across B2 SINE genes (n=3434) with detectable Polr3A peaks (from Fig.1) and expressed during infection (RPKM≥5) (11). Alignment files were averaged from two replicates. ChIP-seq signal was plotted as a histogram with 10 bp bins for -500 to +500bp around the transcription start site (TSS). In the bottom panel, each row of the heat map corresponds to the CPSF30 ChIP-seq signal for each B2 SINE gene from the metaplot above. (C) Polr3A ChIP-seq signals associated with Polr3A occupied peaks at B2 SINEs during infection (from Fig. 1) were plotted against CPSF30-ChIP-seq signals. The correlation (R) between ChIP-seq signals is denoted on the graphs and was calculated using the Pearson correlation coefficient. (D) NIH3T3 murine fibroblasts transfected with control non-targeting or Brf1-targeting siRNAs were mock-treated or infected with MHV68 at an MOI of 5. At 24hpi, cells were harvested and lysed to extract total protein and were analyzed by Western blotting with antibodies against Brf1, CPSF30, and Vinculin (loading control).

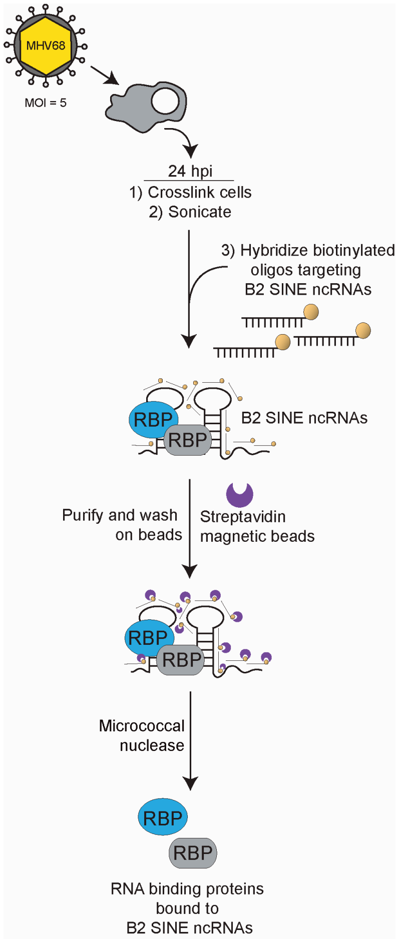

Figure S4. B2 SINE RNAs interact with mRNA 3′-end processing factors during infection
Schematic of the comprehensive identification of RNA-binding proteins (ChIRP) protocol to detect B2 SINE ncRNA-protein interactions during MHV68 infection.

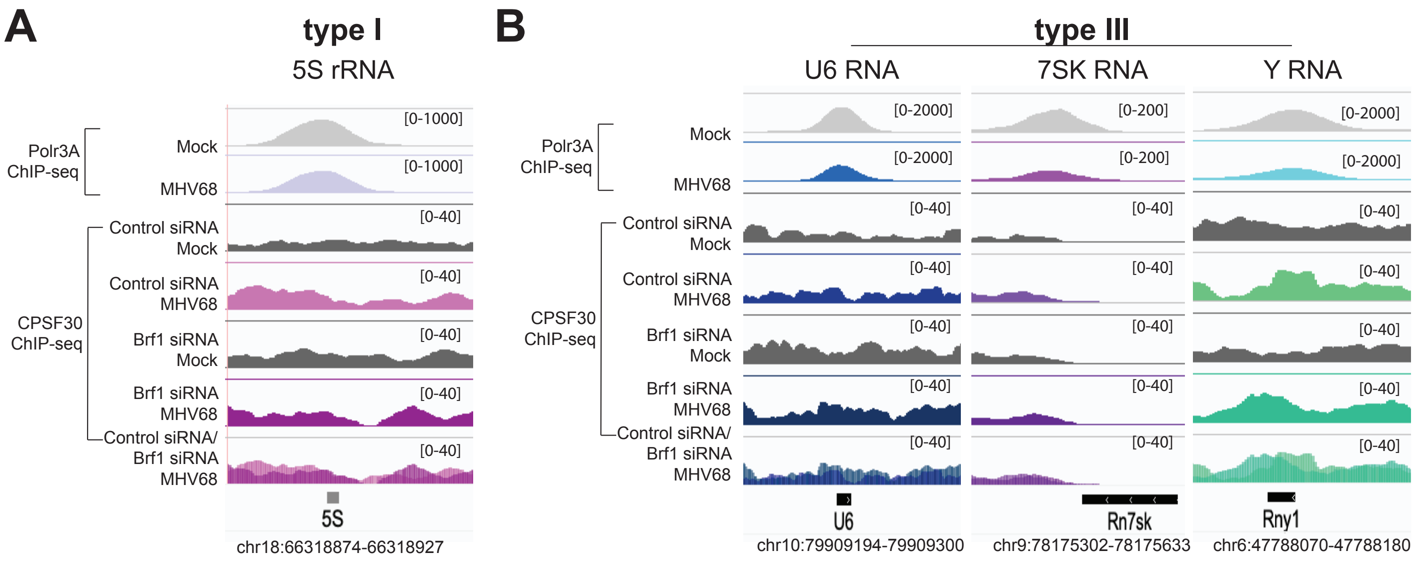

Figure S5. CPSF30 ChIP-seq signal at type I and type III promoters on the cellular genome

Polr3A ChIP-seq coverage across select promoter type I (A) and type III (B) genes from mock-treated or MHV68-infected samples. Alignment files were averaged from two replicates and visualized in the Integrative Genome Viewer (13). Y-axis minimum and maximum values are within brackets.

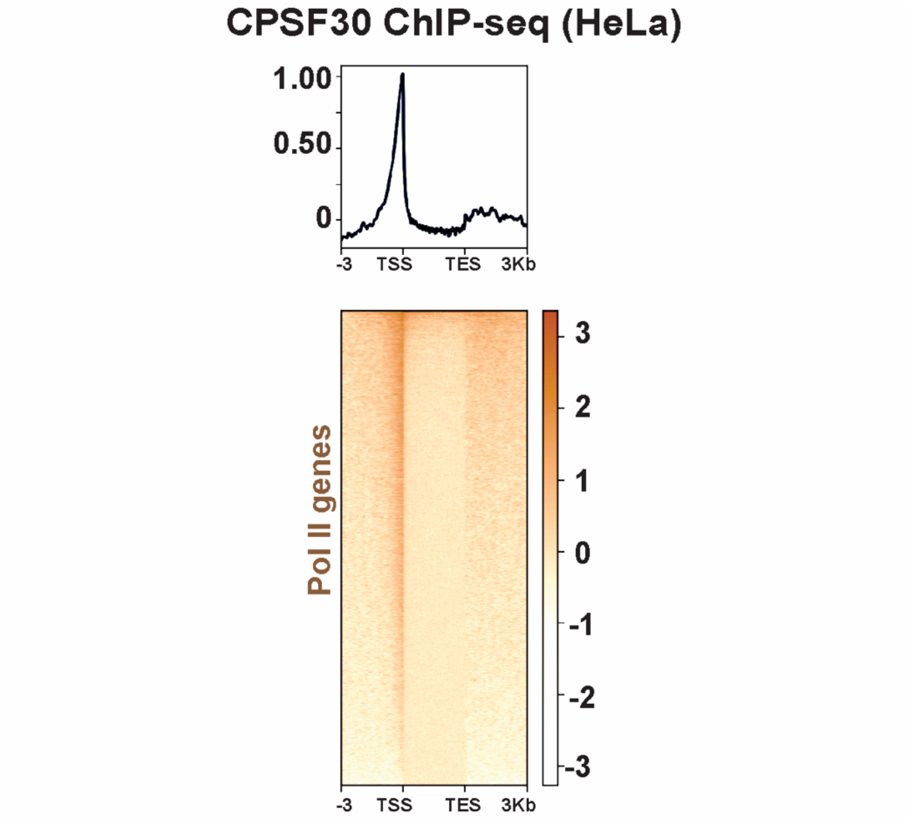

Figure S6. CPSF30 ChIP-seq signal at Pol II transcribed genes

The top panels are metagene plots displaying ChIP-seq signals at Pol II transcribed genes with the highest CPSF30 ChIP-seq signal (n=10000) from previously published CPSF30 ChIP-seq datasets generated from HeLa cells transfected with control antisense morpholino oligonucleotides (14). Alignment files were averaged from two replicates. ChIP-seq signal was plotted as a histogram with 10 bp bins for -3000 to +3000bp around the TSS with 300 bins/gene. In the bottom panel, each row of the heat map corresponds to ChIP-seq signal for Pol II gene from the metaplot above. Heat maps are ranked by CPSF30 ChIP-seq coverage values.

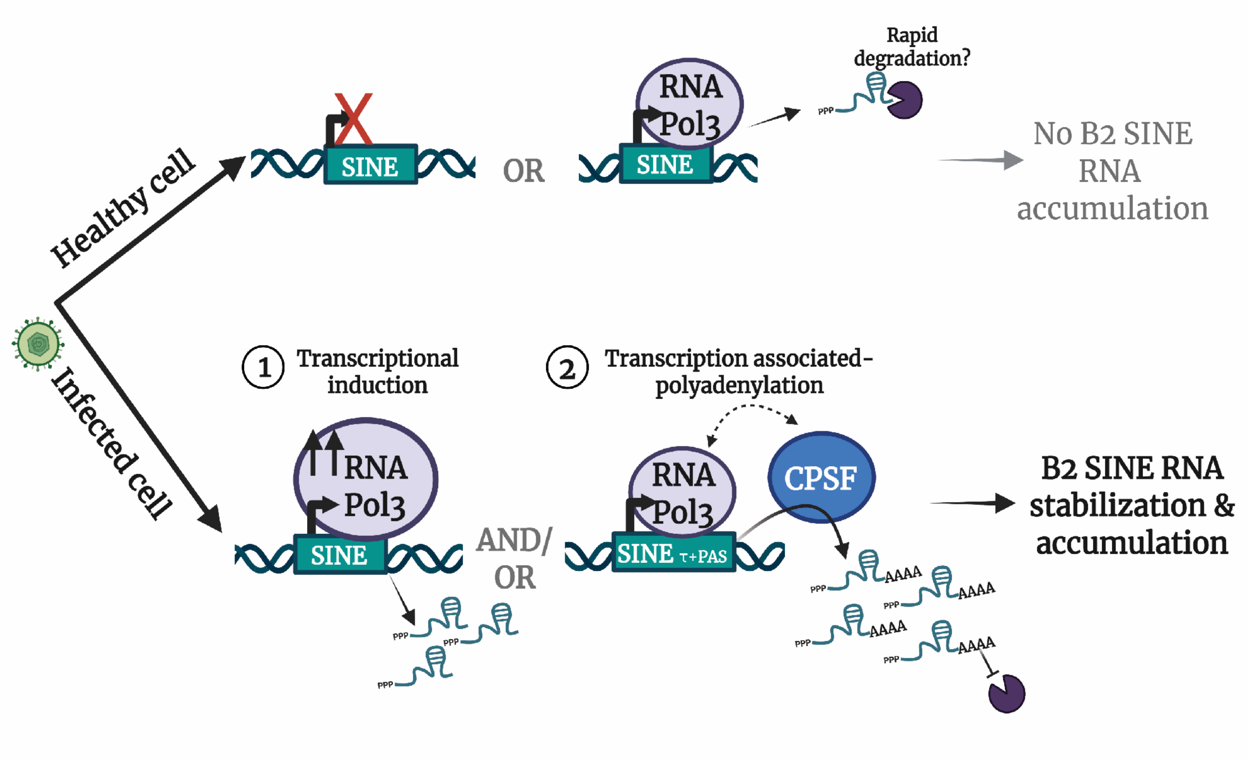

Figure S7. Model of B2 SINE induction and polyadenylation during MHV68 infection
Schematic shows possible models for B2 SINE ncRNA induction during MHV68 infection. The top panel shows B2 SINE loci in healthy cells, which are repressed or occupied by Pol III, with B2 SINE ncRNAs likely being rapidly degraded. The bottom panel shows models for transcriptional induction at B2 SINE genes (1) and polyadenylation of B2 SINE ncRNAs near sites of their transcription via CPSF activity (2), both leading to the accumulation of SINE ncRNAs in virus-infected cells.

Dataset S1 (separate file). Polr3A ChIP-Seq Differential Binding Analysis (MHV68 vs. Mock). DiffBind (7) was used to perform differential binding analysis of Polr3A occupancy data from mock-treated and MHV68-infected samples. The values shown in the dataset are $\log_2$ fold change (FC) (MHV68 vs. Mock) and the false-discovery rate (FDR) *p*-value at locations within the genome found to have peaks using MACS2 (5).

Dataset S2 (separate file). B2 SINE - ChIRP coupled to mass spectrometry (ChIRP-MS). NIH3T3 cells were mock-treated or infected with MHV68 at an MOI of 5. Comprehensive identification of RNA-binding proteins (ChIRP) was performed using biotinylated B2 SINE oligos. Antisense LacZ oligos were used as a negative control. Protein was extracted from ChIRP samples and was subjected to mass-spectrometry. The dataset shows peptide counts and percent coverage detected in the samples.

SI REFERENCES

1. Langmead B, Salzberg SL. 2012. Fast gapped-read alignment with Bowtie 2. Nature Methods 9:357-359.
2. Morrissey A, Shi J, James DQ, Mahony S. 2023. Allo: Accurate allocation of multi-mapped reads enables regulatory element analysis at repeats. bioRxiv doi:10.1101/2023.09.12.556916.
3. Quinlan AR, Hall IM. 2010. BEDTools: a flexible suite of utilities for comparing genomic features. Bioinformatics 26:841-842.
4. Li H, Handsaker B, Wysoker A, Fennell T, Ruan J, Homer N, Marth G, Abecasis G, Durbin R, Subgroup GPDP. 2009. The Sequence Alignment/Map format and SAMtools. Bioinformatics 25:2078-2079.
5. Zhang Y, Liu T, Meyer CA, Eeckhoute J, Johnson DS, Bernstein BE, Nusbaum C, Myers RM, Brown M, Li W, Liu XS. 2008. Model-based Analysis of ChIP-Seq (MACS). Genome Biology 9:R137.
6. Smit A, Hubley, R & Green, P. . 2013-2015. RepeatMasker Open-4.0.
7. Stark RB, G. D. . 2011. DiffBind: differential binding analysis of ChIP-seq peak data.
8. Love MI, Huber W, Anders S. 2014. Moderated estimation of fold change and dispersion for RNA-seq data with DESeq2. Genome Biology 15:550.
9. Huang G, Liu Z, Van Der Maaten L, Weinberger KQ. Densely connected convolutional networks, p 4700-4708. *In* (ed),
10. Ambrosini G, Groux R, Bucher P. 2018. PWMScan: a fast tool for scanning entire genomes with a position-specific weight matrix. Bioinformatics 34:2483-2484.
11. Karijolich J, Zhao Y, Alla R, Glaunsinger B. 2017. Genome-wide mapping of infection-induced SINE RNAs reveals a role in selective mRNA export. Nucleic Acids Research 45:6194-6208.
12. Schindelin J, Arganda-Carreras I, Frise E, Kaynig V, Longair M, Pietzsch T, Preibisch S, Rueden C, Saalfeld S, Schmid B, Tinevez J-Y, White DJ, Hartenstein V, Eliceiri K, Tomancak P, Cardona A. 2012. Fiji: an open-source platform for biological-image analysis. Nature Methods 9:676-682.
13. Robinson JT, Thorvaldsdóttir H, Winckler W, Guttman M, Lander ES, Getz G, Mesirov JP. 2011. Integrative genomics viewer. Nature Biotechnology 29:24-26.
14. Feng Q, Lin Z, Deng Y, Ran Y, Yu R, Xiang AP, Ye C, Yao C. 2023. The U1 antisense morpholino oligonucleotide (AMO) disrupts U1 snRNP structure to promote intronic PCPA modification of pre-mRNAs. J Biol Chem 299:104854.
